# Visualizing and quantifying cerebrospinal venous flow using PC-VIPR

**DOI:** 10.1186/1532-429X-14-S1-W33

**Published:** 2012-02-01

**Authors:** Eric M Schrauben, Kevin Johnson, Aaron Field, Oliver Wieben

**Affiliations:** 1 Medical Physics, University of Wisconsin - Madison, Madison, WI, USA; 2 Department of Radiology, University of Wisconsin - Madison, Madison, WI, USA

## Summary

Presented is a novel MR imaging protocol to comprehensively assess vessel lumen and hemodynamics of the cerebrospinal veins. The 3 station exam is designed to cover middle cerebral, jugular, and azygous veins in order to investigate the CCSVI hypothesis for the association of insufficient venous return with multiple sclerosis.

## Background

Recently, Zamboni et al. have postulated that chronic cerebrospinal venous insufficiency (CCSVI) with reflux to the level of the deep cerebrospinal veins precipitates increased iron deposition and a neuroinflammatory cascade of events that ultimately result in Multiple Sclerosis (MS) (1). He proposed 5 ultrasound (US) based criteria of diameter changes and impaired flow in the cerebrospinal veins to diagnose MS (2), some of which are hard to assess because they require special US hardware and technologist training and US measures are known to be user-dependent. Here we describe our initial experience with a 4D MR Flow approach, PC-VIPR (3), to overcome these problems with a user-independent, non-invasive technique that provides whole vessel coverage.

## Methods

To investigate vessel anatomy and hemodynamics, 7 healthy volunteers were imaged on a clinical 3T system (750 Discovery, GE Healthcare). PC-VIPR of the cerebral, internal jugular (IJV), and azygous veins were performed in a three station exam with a neurovascular phased array and a cardiac coil. Sample imaging parameters for cerebral veins are: Dual Echo, FOV: 24 x 24 x 16 cm, Res: 0.6 x 0.6 x 0.6 mm, 9000 Projections (36x), TR=15.9, BW = 31.25, VENC = 40 cm/s, 7:30 min scan time. Acquisitions were modified to allow for retrospective cardiac gating, with additional modifications being investigated for retrospective double-gated cardiac and respiratory reconstruction to address phasic changes in venous flow due to respiration. A single injection of gadofosveset trisodium (Ablavar, Lantheus Medical Imaging, 0.03-0.05 mmol/kg at 3 ml/s) was used for a first pass perfusion scan, contrast-enhanced MRA, and the PC-VIPR scans.

## Results

**Figure 1:**
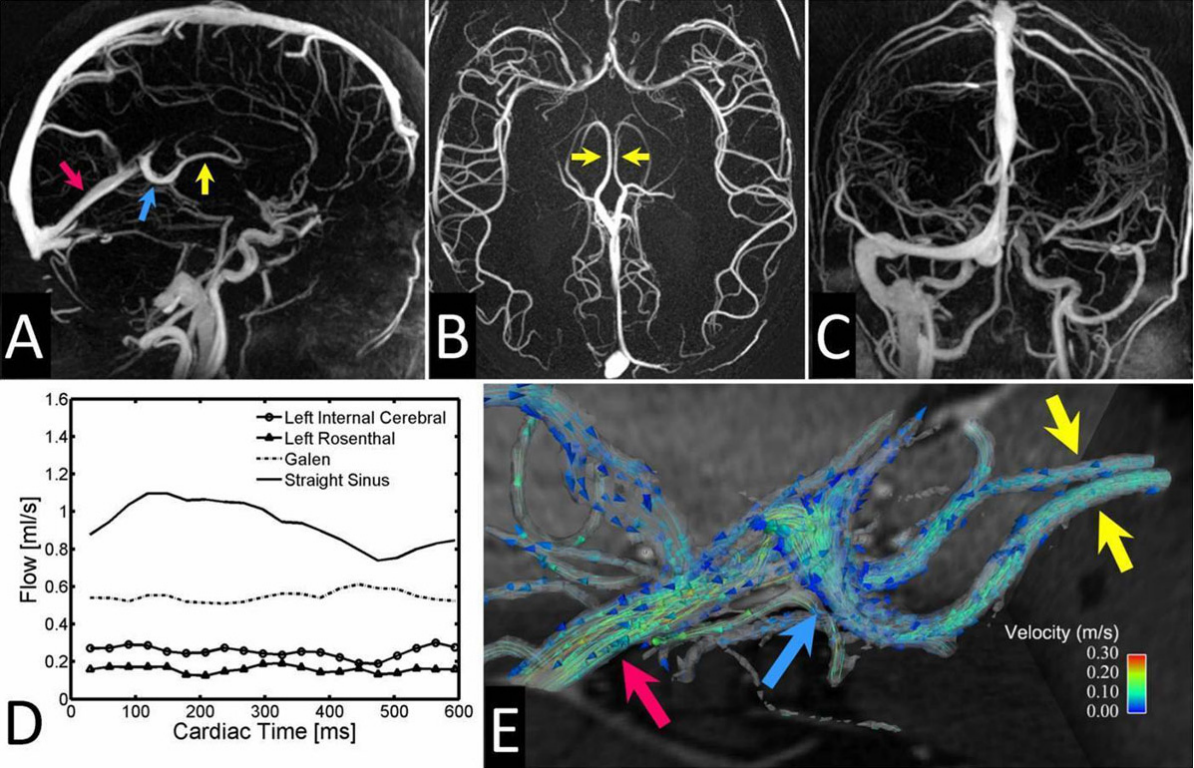
Intracranial PC VIPR results in a healthy volunteer: limited MIP images reformatted in arbitrary orientations without loss of spatial resolution (A-C). Quantitative flow measurements in the deep cerebral veins show only mild variations within the cardiac cycle (D). Corresponding flow visualization in form of vector fields (E). Yellow arrows=internal cerebral veins, blue arrows=vein of Galen, red arrows=straight sinus.

**Figure 2:**
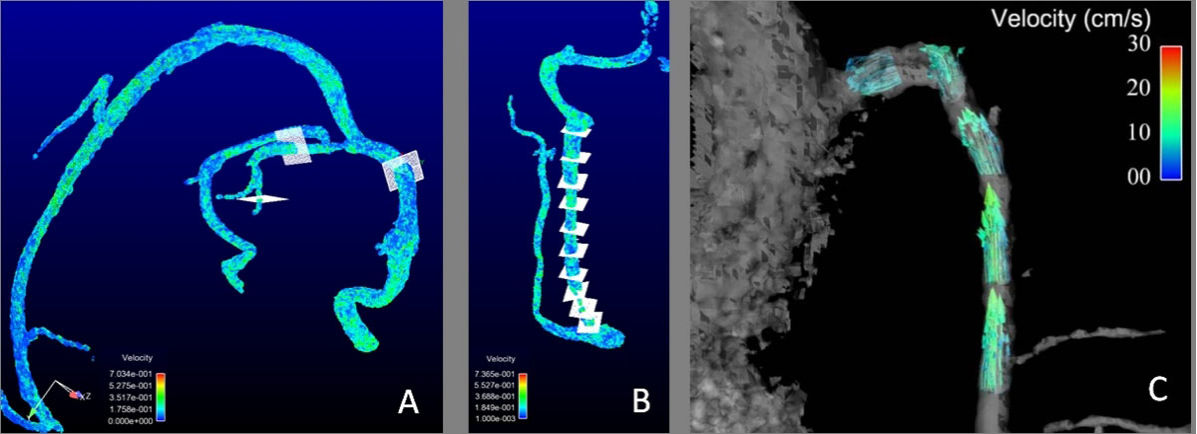
Example visualizations using Ensight software (CEI) of flow for the three stations of the PC-VIPR scans. Planes are placed orthogonal to the direction of flow in the cerebral veins (A), evenly spaced down the IJV (B), and in the azygous vein (C).

Figure 1 shows representative results in the cerebral veins. PC-VIPR allows whole head coverage with high isotropic spatial resolution and visualization of flow characteristics can be collected with retrospective selection of vessels of interest in an arbitrary orientation. Example flow visualizations from the three PC-VIPR sequences are shown in Figure 2. Measurement planes are placed orthogonally to the IJV, from which reflux over the cardiac cycle can be easily detected and quantified.

## Conclusions

This feasibility study demonstrates the potential for 4D MR flow acquisitions to noninvasively measure and visualize vessel anatomy and velocity fields in the cerebrospinal veins. The use of PC VIPR provides hemodynamic information over a large vascular territory, thereby greatly simplifying scan prescription and providing more complete information along vessel paths as compared to multiple 2D PC acquisitions.
